# Molecular and metabolic features of colistin-resistant ST11 carbapenem-resistant Klebsiella pneumoniae

**DOI:** 10.1099/jmm.0.002217

**Published:** 2026-11-03

**Authors:** Ling Hao, Xiao Yang, Nadeem Ullah

**Affiliations:** 1 Department of Pulmonary and Critical Care Medicine, Guangzhou First People's Hospital, School of Medicine, South China University of Technology, Guangzhou, PR China; 2 Institute of Clinical Medicine, Guangzhou First People's Hospital, School of Medicine, South China University of Technology, Guangzhou, PR China; 3 Department of Laboratory Medicine, Guangzhou First People's Hospital, School of Medicine, South China University of Technology, Guangzhou, PR China; 4 Department of Clinical Microbiology, Umeå University, Umeå, Sweden

**Keywords:** biofilm, carbapenem-resistant, colistin resistance, *Klebsiella pneumoniae*, lipid metabolism, untargeted metabolomics, virulence

## Abstract

**Introduction.** The emergence of colistin-resistant carbapenem-resistant *Klebsiella pneumoniae* (CRKP) poses a critical threat to public health, a challenge that was exacerbated by the coronavirus disease 2019 (COVID-19) pandemic.

**Gap statement.** The specific metabolic adaptations that underlie colistin resistance in high-risk sequence type 11 (ST11) CRKP clinical strains, particularly in direct comparison with their susceptible counterparts, remain poorly characterized.

**Aim.** To explore metabolic features associated with colistin resistance in clinical CRKP isolates.

**Methodology.** Twelve CRKP strains, encompassing both colistin-resistant and colistin-susceptible isolates, were analysed for antimicrobial resistance profiles, virulence-associated genes, sequence types and biofilm formation capacity. Comparative untargeted metabolomics was performed to map metabolic shifts between the resistant and susceptible groups.

**Results.** All isolates belonged to the high-risk clone ST11, harboured the KPC-2 carbapenemase and carried multiple virulence genes, with K2 being the dominant serotype. All isolates formed biofilms, with resistant isolates showing enhanced capacity. Colistin resistance was chromosomally mediated by *pmrB* and *mgrB* mutations, with no plasmid-encoded *mcr* genes detected. Untargeted metabolomics identified 126 significantly differentially expressed metabolites (DEMs) between resistant and susceptible isolates. These DEMs were significantly enriched in lipids and lipid-like molecules. Colistin-resistant strains exhibited increased glycerophospholipids, sphingolipids and fatty acids, coupled with decreased metabolites from central carbon and amino acid pathways.

**Conclusion.** In this exploratory study, colistin resistance in these ST11 CRKP strains was associated with chromosomal alterations in *pmrB* and *mgrB* and with marked shifts in membrane-associated lipid metabolites. While these metabolic changes suggested membrane remodelling, they remain associative and require functional validation. Beyond resistance, the co-carriage of these traits with robust virulence gene expression highlights a critical clinical challenge, supporting the need for further research into combination therapies targeting these adaptive lipid pathways.

## Data Summary

The data used to support the findings of this study are available from the corresponding author upon request.

## Introduction

*Klebsiella pneumoniae* (*K. pneumoniae*), a member of the ESKAPE pathogens (*Enterococcus faecium*, *Staphylococcus aureus*, *Klebsiella pneumoniae*, *Acinetobacter baumannii*, *Pseudomonas aeruginosa* and *Enterobacter* spp.), is a major cause of both opportunistic nosocomial and community-acquired infections [1]. Most *K. pneumoniae* clinical isolates exhibit multidrug resistance (MDR) and can infect diverse human body sites, causing pneumonia, meningitis, liver abscesses, bacteremia, urinary tract infections, soft tissue infections and surgical-site infections [1]. The emergence and rapid global spread of carbapenem-resistant *K. pneumoniae* (CRKP) pose a serious public health threat, compounded by limited therapeutic options, high treatment costs and poor patient outcomes [2]. The majority of CRKP clinical isolates are resistant to nearly all available antibiotics and exhibit high virulence [3], contributing to mortality rates potentially exceeding 37% in severe cases, even with adequate treatment [4]. In China, *K. pneumoniae* is a leading cause of respiratory tract infections [5]. The national resistance rates to carbapenems have increased alarmingly, rising more than sevenfold since 2005 to ∼21% for both meropenem and imipenem by 2025 [5].

Colistin is a last-resort antibiotic for life-threatening infections caused by carbapenem-resistant Gram-negative bacteria [6]. However, the widespread and often unnecessary use of colistin has driven the emergence of colistin-resistant strains [7]. Following the COVID-19 pandemic, colistin-resistant *K. pneumoniae* isolates were identified in the respiratory department of our hospital [8]. Thus, a deeper understanding of the mechanism underlying colistin resistance in *K. pneumoniae* is urgently needed to support future surveillance, therapeutic and infection-control strategies against this emerging resistance.

The primary mechanisms of colistin resistance involve chromosomal or plasmid-mediated alterations, including modifications in the two-component regulatory systems PmrA/PmrB and PhoP/PhoQ, mutations in the *mgrB* gene and acquisition of plasmid-encoded *mcr* genes [9–11]. In addition to these genetic mechanisms, bacterial metabolic states may influence antimicrobial tolerance and adaptation. This is evidenced by amino acid metabolism, which confers tolerance to environmental stress through mechanisms, such as intracellular pH homeostasis, energy production and motility reduction [12]. Furthermore, carbohydrate metabolism contributes to biofilm formation and maintenance, which in turn drives the evolution of drug resistance [13]. A previous proteomic analysis has identified differential protein expression between colistin-susceptible and colistin-resistant *K. pneumoniae* strains, with enrichment in metabolic processes, including amino sugar and nucleotide sugar metabolism, propanoate metabolism and phenylalanine metabolism [14]. To explore metabolic features associated with colistin resistance, we performed untargeted metabolomics on twelve CRKP strains, including colistin-resistant and colistin-susceptible isolates. These strains were also characterized for their antimicrobial resistance profiles, virulence-associated genes, sequence types (STs) and biofilm formation capacity.

## Methods

### Bacterial strains and culture

Twelve CRKP strains were isolated from the clinical samples of inpatients in our hospital. Half of these strains (MDR1 to MDR6) were susceptible to colistin, while the others (XDR1 to XDR6) were colistin-resistant. All isolates were identified as *K. pneumoniae* using biochemical tests and the IVD MALDI Biotype system (Bruker Daltonics, GmbH and Co., Bremen, Germany). All isolates were cultured in LB broth to mid-log phase (OD_600_ values=0.4–0.6), washed twice with cold PBS, harvested and quenched in liquid nitrogen for 15 min to stop the metabolic process. The samples were stored at −80 °C before metabolomics analysis.

### Antimicrobial susceptibility testing

Routine antimicrobial susceptibility testing was examined using a Vitek-2 Compact automatic microbiology system (bioMérieux, Inc., Marcy-l’Étoile, France). The susceptibility of all strains against colistin was further confirmed by the broth microdilution method according to the guideline recommended by the Clinical and Laboratory Standards Institute (CLSI) as described previously [15, 16]. *Escherichia coli* ATCC 25922 and *Pseudomonas aeruginosa* ATCC 27853 were used as the control organisms.

### Multilocus sequence typing analysis

Multilocus sequence typing (MLST) was performed on all *K. pneumoniae* isolates as described previously [17]. Briefly, seven conserved housekeeping genes (*gapA*, *infB*, *mdh*, *pgi*, *phoE*, *rpoB* and *tonB*) were amplified and sequenced, and the sequences were analysed using the online database on the Pasteur Institute MLST website (http://bigsdb.pasteur.fr/klebsiella/klebsiella.html, accessed on 19 April 2023). The STs were identified according to the allelic profiles in the database.

### Carbapenemase detection

The 3-aminophenylboronic acid (APB)/EDTA combined disc test was used to detect carbapenemases and to differentiate between class A carbapenemases and metallo-β-lactamases [18]. The test was performed by inoculating Mueller-Hinton agar as given for the standard diffusion method and placing onto the agar an imipenem disc alone, and three discs of imipenem containing 300 µg APB, 292 µg EDTA or both 300 µg APB and 292 µg EDTA. The agar plates were incubated at 37 °C overnight. The diameter of the growth-inhibitory zone around the imipenem discs with APB, EDTA or APB plus EDTA was compared with that around the plain imipenem disc.

### Molecular detection of antibiotic resistance and Virulence-Associated genes

The genomic DNA of each isolate was extracted using a DNA extraction kit (Tiangen, Beijing, China) according to the manufacturer’s protocol. The presence of carbapenemases (*bla*_KPC-2_, *bla*_NDM_, *bla*_VIM_, *bla*_IMP_ and *bla*_OXA-48_ type), and extended-spectrum β-lactamases associated genes (*bla*_TEM_, *bla*_SHV_ and *bla*_CTX-M_) was analysed by conventional PCR using validated primers and methods, and the amplified products were confirmed by sequencing [19, 20]. The extensively drug-resistant (XDR) strains were further examined for the presence of plasmid-mediated mobile colistin resistance genes (*mcr-1* to *mcr-8*) and chromosomal mutations in *mgrB*, *phoP*/*phoQ*, *pmrA* and *pmrB* [21–23]. The prevalence of fourteen virulence-associated genes – including *iucA*, *iroB*, *peg-344*, *_p_rmpA*, *_p_rmpA2*, *magA*, *fimH*, *entB*, *irp2*, *ureA*, *iutA*, *wabG*, *uge* and *mrkD* – was determined by PCR using established primers and methods [24–27]. Capsular serotypes (K1, K2, K5, K20, K54 and K57) were also detected by PCR [28]. All analyses were performed with corresponding positive controls. All primer sequences were listed in Table 1.

**Table 1.** The sequences of primers used in this study

Gene	Forward primer (5′→3′)	Reverse primer (5′→3′)	Fragment (bp)	Reference
*bla*_KPC-2_	CGTCTAGTTCTGCTGTCTTG	CTTGTCATCCTTGTTAGGCG	798	[19]
*bla*_NDM_	GGTTTGGCGATCTGGTTTTC	CGGAATGGCTCATCACGATC	621	[19]
*bla*_OXA-48_	GCGTGGTTAAGGATGAACAC	CATCAAGTTCAACCCAACCG	438	[19]
*bla*_IMP_	GGAATAGAGTGGCTTAAYTCTC	GGTTTAAYAAAACAACCACC	232	[19]
*bla*_VIM_	GATGGTGTTTGGTCGCATA	CGAATGCGCAGCACCAG	390	[19]
*bla*_TEM_	GGGTTATTCTTATTTGTCGCT	TAGCGTTGCCAGTGCTCG	929	[20]
*bla*_SHV_	AAAATTCTTGAACG	TTACCAAATGCTTAATCA	1080	[20]
*bla*_CTX-M_	TTTGCGATGTGCAGTACCAGTAA	CGATATCGTTGGTGGTGCCATA	544	[20]
*mcr-1*	ATCAGCCAAACCTATCCCATCG	GCAGACGCACAGCAATGCCTAT	1257	[21]
*mcr-2*	GCGATGGCGGTCTATCCTGTAT	TGCGATGACATGGGGTGTCAGC	378	[21]
*mcr-3*	TATGGGTTACTATTGCTGG	CTACCCTGATGCTCATCG	814	[21]
*mcr-4*	GTCATAGTGGTATAAAAGTACAG	CCACCGTCTATCAGAGCCAAC	669	[21]
*mcr-5*	GCGGTTGTCTGCATTTATCAC	CTTTGAAAACCTGTCTTCGGCA	1042	[21]
*mcr-6*	GTCCGGTCAATCCCTATCTGT	ATCACGGGATTGACATAGCTAC	556	[21]
*mcr-7*	TGCTCAAGCCCTTCTTTTCGT	TTCATCTGCGCCACCTCGT	892	[21]
*mcr-8*	AACCGCCAGAGCACAGAATT	TTCCCCCAGCGATTCTCCAT	667	[21]
*mgrB*	TTAAGAAGGCCGTGCTATCC	AAGGCGTTCATTCTACCACC	253	[22]
*phoP*	CCATGCAGTATGCGCAAAGG	GCTAACGCTATAGCCCACCA	950	[23]
*phoQ*	ATACCCACAGGACGTCATCA	CAGGTGTCTGACAGGGATTA	1597	[22]
*pmrA*	CATTTCCGCGCACTGTCTGC	CAGGTTTCAGTTGCAAACAG	851	[22]
*pmrB*	ACCTACGCGAAAAGATTGGC	GATGAGGATAGCGCCCATGC	1274	[22]
*iucA*	GCTTATTTCTCCCCAACCC	TCAGCCCTTTAGCGACAAG	583	[24]
*iroB*	CAAAAAAGCAGCAGAGGC	TCACTGGCGGAATCCAACAC	585	[24]
*peg-344*	AAAGGACAGAAAGCCAGTG	CAATGACGAGGGGGATAATC	411	[24]
*_p_rmpA*	GAGTAGTTAATAAATCAATAGCAAT	CAGTAGGCATTGCAGCA	332	[24]
*_p_rmpA2*	GTGCAATAAGGATGTTACATTA	GGATGCCCTCCTCCTG	430	[24]
*magA*	GGTGCTCTTTACATCATTGC	GCAATGGCCATTTGCGTTAG	1282	[25]
*fimH*	TGCTGCTGGGCTGGTCGATG	GGGAGGGTGACGGTGACATC	909	[25]
*entB*	ATTTCCTCAACTTCTGGGGC	AGCATCGGTGGCGGTGGTCA	371	[25]
*irp2*	GCTACAATGGGACAGCAACGAC	GCAGAGCGATACGGAAAATGC	230	[26]
*ureA*	GACAAGCTGTTGCTGTTTACC	CGGGTTGTGAACGGTGAC	270	[25]
*iutA*	GGCTGGACATCATGGGAACTGG	CGTCGGGAACGGGTAGAATCG	300	[25]
*wabG*	ACCATCGGCCATTTGATAGA	CGGACTGGCAGATCCATATC	683	[25]
*uge*	TCTTCACGCCTTCCTTCACT	GATCATCCGGTCTCCCTGTA	534	[25]
*mrkD*	TATTGGCTTAATGGCGCTGG	TAATCGTACGTCAGGTTAAAGACC	920	[27]
K1	GGTGCTCTTTACATCATTGC	GCAATGGCCATTTGCGTTAG	1283	[28]
K2	GACCCGATATTCATACTTGACAGAG	CCTGAAGTAAAATCGTAAATAGATGGC	641	[28]
K5	TGGTAGTGATGCTCGCGA	CCTGAACCCACCCCAATC	280	[28]
K20	CGGTGCTACAGTGCATCATT	GTTATACGATGCTCAGTCGC	741	[28]
K54	CATTAGCTCAGTGGTTGGCT	GCTTGACAAACACCATAGCAG	881	[28]
K57	CTCAGGGCTAGAAGTGTCAT	CACTAACCCAGAAAGTCGAG	1037	[28]

### String test

Colonies of the isolates cultured overnight on Columbia blood agar plates were stretched outward using an inoculation loop, as described previously [14]. The result was considered positive if a viscous string formation exceeded 5 mm in length.

### Biofilm formation assay

Biofilm formation was performed using the crystal violet staining method as published and described previously [14]. The tests were performed in triplicate, and the results were averaged and interpreted based on the criteria of a previous report [29].

### Metabolites extraction

Metabolites were extracted by treating samples with ice-cold 80% methanol, followed by dual agitation: 30 s of vortexing and 6 min of ultrasonication maintained in an ice-water bath. Supernatants were isolated via centrifugation (12,000 r.p.m., 15 min, 4 °C). These aliquots were subsequently lyophilized and redissolved in 10% methanol before analytical testing. Quality control (QC) samples were prepared by mixing equal supernatant volumes from all biological replicates.

### Ultra-high performance liquid chromatography-MS/MS analysis

The sample analysis was conducted using a liquid chromatography-mass spectrometry platform (Vanquish ultra-high performance liquid chromatography (UHPLC) coupled to an Orbitrap Q Exactive^TM^ HF-X mass spectrometer, Thermo Fisher, Novogene Co.,Ltd., Beijing, China). For separation, a Hypersil Gold C18 column (2.1×100 mm, 1.9 µm) was employed at a flow rate of 0.2 ml min^−1^. The gradient programme consisted of an initial 1.5 min hold at 2% solvent B, a ramp from 2–85% B over 3 min, an increase to 100% B over 10 min, rapid return to 2% B at 10.1 min, followed by re-equilibration at 2% B for a total run time of 23 min. Mobile phases were: Eluent A (0.1% formic acid for positive mode; 5 mM ammonium acetate at pH 9.0 for negative mode) and Eluent B (methanol). The mass spectrometer alternated between positive and negative ionization modes, operating at a 3.5 kV spray voltage, 320 °C capillary temperature and gas flows of 35 (sheath) and 10 (auxiliary) arbitrary units, with the S-lens RF level set to 60 and the auxiliary gas heater at 350 °C.

### Data processing, bioinformatic and statistical analysis

Metabolomics data processing began with peak alignment and quantification via Compound Discoverer 3.3 (Thermo Fisher), normalized by total spectral intensity. Metabolite identification involved predicting molecular formulas and matching against the mzCloud database (https://www.mzcloud.org/, accessed July 17, 2023) and in-house libraries (mzVault/MassList). Further annotation of QC-filtered metabolites (coefficient of variation ≤30%) was achieved using Kyoto Encyclopedia of Genes and Genomes (KEGG) (http://www.genome.jp/kegg/) and ClassyFire (http://classyfire.wishartlab.com/). Discriminant analysis was performed using PLS-DA to obtain variable importance in the projection (VIP) scores. Statistically significant metabolites were defined by univariate t-tests (*P*<0.05) alongside VIP >1 and fold change (FC) >1.5 or <0.667. These filtered metabolites allowed for a robust comparison between resistant and susceptible isolates. Downstream visualization (Volcano plot and hierarchical clustering) and pathway enrichment analysis were executed using R packages and the KEGG database, respectively.

## Results

### Antimicrobial susceptibility profile and resistance mechanism of colistin-resistant CRKP isolates

The antimicrobial resistance profile showed that all CRKP isolates in this study exhibited extensive resistance to nearly all tested antibiotic classes but remained sensitive to tigecycline (Table 2). The MIC values of the XDR strains against colistin ranged from 16 to 64 µg ml^−1^ (Table 3). All strains were determined to be carbapenemase producers via the APB/EDTA method (data not shown). In line with the phenotypic results, all isolates were positive for *bla*_KPC-2_ and harboured both *bla*_SHV_ and *bla*_CTX-M_ (Table 3). The colistin-susceptible MDR isolates revealed WT *pmrA*, *pmrB, phoP*, *phoQ* and *mgrB* sequences. The colistin-resistant XDR isolates exhibited a chromosomally mediated mechanism of colistin resistance, and plasmid-encoded *mcr* genes were not detected in any of these strains (Table 3). The XDR1 strain had a point mutation in the coding region of *pmrB*: GAC (codon 150, aspartic acid) converted to TAC (tyrosine). The XDR2 strain had *mgrB* premature termination: TGG (codon 20, tryptophan) converted to TGA (stop codon). The other four isolates (XDR3 to XDR6) showed larger *mgrB* amplicons (1,210 bp) through PCR amplification. These alterations were related to IS elements belonging to the IS5-like family, which were inserted at bp 70 within the coding region of the *mgrB* gene.

**Table 2.** The antimicrobial resistance profile of the MDR and XDR isolates

Antibiotic	MDR1	MDR2	MDR3	MDR4	MDR5	MDR6	XDR1	XDR2	XDR3	XDR4	XDR5	XDR6
Amikacin	R	S	S	S	S	S	R	R	R	R	R	R
Aztreonam	R	R	R	R	R	R	R	R	R	R	R	R
Cefepime	R	R	R	R	R	R	I	R	R	R	R	R
Cefoperazone/Sulbactam	R	R	R	R	R	R	R	R	R	R	R	R
Ceftazidime	R	R	R	R	R	R	R	R	R	R	R	R
Ciprofloxacin	R	R	R	R	R	R	R	R	R	R	R	R
Colistin	S	S	S	S	S	S	R	R	R	R	R	R
Doxycycline	R	R	R	R	R	R	R	R	R	S	R	R
Imipenem	R	R	R	R	R	R	R	R	R	R	R	R
Levofloxacin	R	R	R	R	R	R	R	R	R	R	R	R
Meropenem	R	R	R	R	R	R	R	R	R	R	R	R
Minocycline	R	R	R	I	R	R	S	R	R	R	R	R
Piperacillin/Tazobactam	R	R	R	R	R	R	R	R	R	R	R	R
Ticarcillin/Clavulanic acid	R	R	R	R	R	R	R	R	R	R	R	R
Tigecycline	S	S	S	S	S	S	S	S	S	S	S	S
Tobramycin	R	R	R	R	R	R	R	R	R	R	R	R
Trimethoprim/Sulfa	R	S	S	S	S	S	S	R	S	R	R	R

R, resistant.I, intermediate; S, susceptible.

**Table 3.** Resistance and virulence gene profiles of the MDR and XDR isolates

Strain	MIC of colistin	Mechanism of colistin resistance	Resistance gene	Virulence gene	Serotype
MDR1	1	–	*bla*_KPC-2_, *bla*_SHV_, *bla*_CTX-M_	*iucA*, *iroB*, *peg-344*, *_p_rmpA*, *_p_rmpA2*, *fimH*, *entB*, *ureA*, *iutA*, *wabG*, *uge*, *mrkD*	K20
MDR2	0.5	–	*bla*_KPC-2_, *bla*_SHV_, *bla*_CTX-M_	*peg-344*, *_p_rmpA*, *_p_rmpA2*, *fimH*, *entB*, *irp2*, *ureA*, *iutA*, *wabG*, *uge*	K2
MDR3	2	–	*bla*_KPC-2_, *bla*_SHV_, *bla*_CTX-M_	*iucA*, *_p_rmpA2*, *fimH*, *entB*, *ureA*, *wabG*, *uge*, *mrkD*	K2
MDR4	0.5	–	*bla*_KPC-2_, *bla*_SHV_, *bla*_CTX-M_	*iucA*, *peg-344*, *_p_rmpA*, *_p_rmpA2*, *fimH*, *entB*, *ureA*, *iutA*, *wabG*, *uge*, *mrkD*	N
MDR5	0.5	–	*bla*_KPC-2_, *bla*_SHV_, *bla*_CTX-M_	*_p_rmpA*, *_p_rmpA2*, *fimH*, *entB*, *ureA*, *wabG*, *uge*, *mrkD*	K2
MDR6	2	–	*bla*_KPC-2_, *bla*_SHV_, *bla*_CTX-M_	*iucA*, *iroB*, *fimH*, *entB*, *irp2*, *ureA*, *iutA*, *wabG*, *uge*, *mrkD*	N
XDR1	64	*pmrB*	*bla*_KPC-2_, *bla*_SHV_, *bla*_CTX-M_	*iroB*, *peg-344*, *_p_rmpA2*, *fimH*, *entB*, *irp2*, *ureA*, *iutA*, *wabG*, *uge*, *mrkD*	N
XDR2	16	*mgrB*	*bla*_KPC-2_, *bla*_SHV_, *bla*_CTX-M_	*iucA*, *iroB*, *_p_rmpA*, *_p_rmpA2*, *fimH*, *entB*, *irp2*, *ureA*, *iutA*, *wabG*, *uge*, *mrkD*	K2
XDR3	32	*mgrB*	*bla*_KPC-2_, *bla*_SHV_, *bla*_CTX-M_	*iroB*, *peg-344*, *_p_rmpA*, *_p_rmpA2*, *fimH*, *entB*, *irp2*, *ureA*, *iutA*, *wabG*, *uge*	K2
XDR4	32	*mgrB*	*bla*_KPC-2_, *bla*_SHV_, *bla*_CTX-M_	*iroB*, *peg-344*, *_p_rmpA2*, *fimH*, *entB*, *irp2*, *ureA*, *iutA*, *wabG*, *uge*, *mrkD*	N
XDR5	16	*mgrB*	*bla*_KPC-2_, *bla*_SHV_, *bla*_CTX-M_	*iucA*, *iroB*, *peg-344*, *_p_rmpA*, *_p_rmpA2*, *fimH*, *entB*, *irp2*, *ureA*, *iutA*, *wabG*, *uge*, *mrkD*	K2
XDR6	16	*mgrB*	*bla*_KPC-2_, *bla*_SHV_, *bla*_CTX-M_	*iucA*, *iroB*, *peg-344*, *_p_rmpA*, *_p_rmpA2*, *fimH*, *entB*, *irp2*, *ureA*, *iutA*, *wabG*, *uge*, *mrkD*	N

N: Nontypable.

### Detection of virulence genes

As shown in Table 3, all isolates acquired at least two of the five key virulence genes (*iucA*, *iroB*, *peg-344*, *_p_rmpA* and *_p_rmpA2*), which were reported to be biomarkers that distinguish hypervirulent *K. pneumoniae* from classical *K. pneumoniae* [24]. Other virulence genes, including *fimH*, *entB*, *ureA*, *wabG* and *uge* were detected in all strains. However, no isolates harboured *magA*, which is related to the K1 capsule serotype. Six isolates (MDR2, MDR3, MDR5, XDR2, XDR3 and XDR5) belonged to capsular serotype K2, and one isolate (MDR1) belonged to K20. The remaining five isolates were not typeable using the PCR targets included in this study and were, therefore, classified as K-nontypable.

### MLST and biofilm forming capacity

MLST analysis revealed that all strains belonged to ST11 (Table 4). Only the MDR1 strain demonstrated a positive string test result. *In vitro* biofilm formation assays indicated that all isolates were biofilm producers. The MDR strains were categorized as strong, moderate or weak biofilm producers (two cases each). Among the XDR strains, four were strong and two were moderate. The OD_590_ nm values of the MDR and XDR strains were 0.825±0.510 and 1.510±0.677, respectively. Because all isolates belonged to ST11, higher-resolution genomic analysis would be required to determine whether biofilm differences reflect resistance status, clonal background or isolate-specific variation.

**Table 4.** MLST and molecular characteristics of CRKP isolates

Strain	Housekeeping gene							ST	String test	Biofilm-forming capacity	Quantitative biofilm formation
	*gapA*	*infB*	*mdh*	*pgi*	*phoE*	*rpoB*	*tonB*				
MDR1	3	3	1	1	1	1	4	11	+	Strongly adherent	1.362
MDR2	3	3	1	1	1	1	4	11	−	Moderately adherent	0.918
MDR3	3	3	1	1	1	1	4	11	−	Strongly adherent	1.457
MDR4	3	3	1	1	1	1	4	11	−	Weakly adherent	0.299
MDR5	3	3	1	1	1	1	4	11	−	Weakly adherent	0.289
MDR6	3	3	1	1	1	1	4	11	−	Moderately adherent	0.623
XDR1	3	3	1	1	1	1	4	11	−	Strongly adherent	1.171
XDR2	3	3	1	1	1	1	4	11	−	Moderately adherent	1.017
XDR3	3	3	1	1	1	1	4	11	−	Strongly adherent	2.239
XDR4	3	3	1	1	1	1	4	11	−	Strongly adherent	1.905
XDR5	3	3	1	1	1	1	4	11	−	Moderately adherent	0.588
XDR6	3	3	1	1	1	1	4	11	−	Strongly adherent	2.138

String test: +, positive; −, negative (positive defined as string length >5 mm). Quantitative biofilm formation was determined by OD_590_ measurement. Biofilm-forming capacity was categorized as strongly, moderately, or weakly adherent based on the OD cutoff criteria in the reference.

MLST, multilocus sequence typing.

### The metabolic spectra of colistin-resistant and -susceptible isolates

Untargeted metabolomics based on UHPLC-MS/MS technology was performed to identify key metabolites associated with colistin resistance in *K. pneumoniae*. In the present study, a total of 1023 metabolites were identified in the MDR and XDR isolates, of which 635 were in positive mode and 388 were in negative mode. The PLS-DA score plot showed visible separation between the MDR and XDR groups in both positive and negative modes (Fig. 1a). Permutation tests validated that the model was not overfitted (Fig. 1b), indicating reliable interpretability and good predictability of the data.

**Fig. 1.**
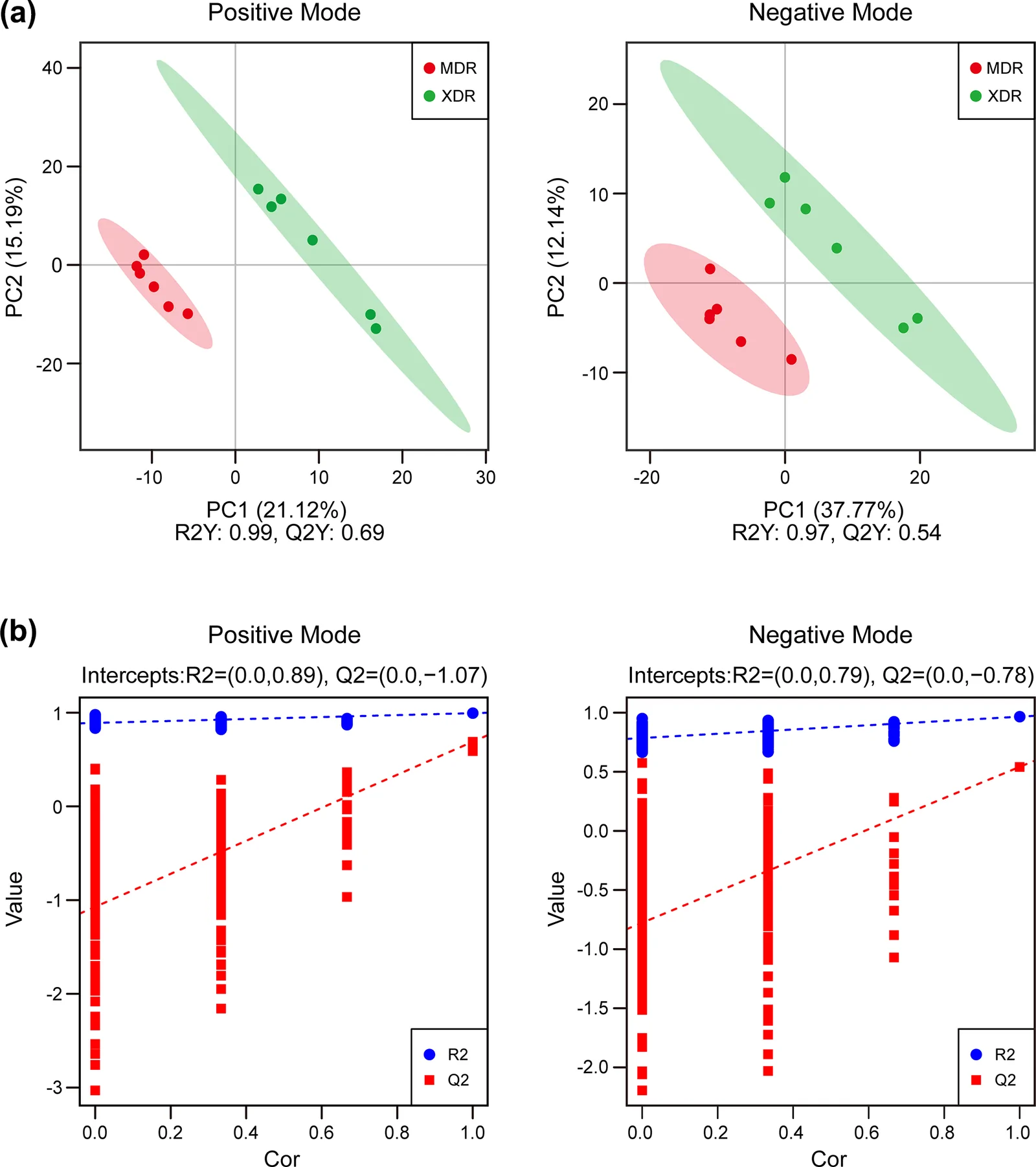
PLS-DA of the metabolites identified in the MDR and XDR groups under the positive and negative ion modes. (**a**) PLS-DA score plot. (**b**) PLS-DA permutation plot.

### Identification of differentially expressed metabolites associated with colistin resistance

Metabolites with differences between the XDR and MDR strains were determined based on VIP, FC and *P*-values. As shown in Fig. 2a, there were 126 differentially expressed metabolites (DEMs), including 80 up-regulated metabolites (VIP >1, FC >1.5, *P<*0.05) and 46 down-regulated metabolites (VIP >1, FC <0.67, *P<*0.05). Metabolic patterns of the DEMs between the XDR and MDR groups were illustrated in the heatmap (Fig. 2b). Details of these DEMs are demonstrated in Table S1 (available in the online Supplementary Material). All altered metabolites were grouped based on the ClassyFire database, which were assigned to benzenoids, lipids and lipid-like molecules, nucleosides/nucleotides/analogues, organic acids/derivatives, organic nitrogen compounds, organic oxygen compounds, organoheterocyclic compounds, organosulfur compounds, phenylpropanoids and polyketides and undefined metabolites. Lipids and lipid-like molecules represented the most prominently altered class (Fig. 2c). KEGG pathway analysis showed that 12 differential metabolites (five were up-regulated and seven were down-regulated) between the XDR and MDR groups participated in different biochemical metabolic pathways (Table 5). In addition, the metabolic regulatory network was constructed, including 24 compounds, 30 enzymes, 15 modules, 91 reactions and 8 pathways (Fig. S1).

**Table 5.** The differential metabolites involved in the KEGG pathways

Metabolite	Formula	Mass	Type	VIP	*P*-value	**Fold change**	KEGG pathway
Palmitoleic acid	C_16_H_30_O_2_	276.209	up	1.846	0.004	1.849	Fatty acid (FA) biosynthesis
S-Adenosyl-l-methionine	C_15_H_22_N_6_O_5_S	398.139	up	2.053	0.002	4.476	Sulphur relay system; Biosynthesis of amino acids; Monobactam biosynthesis; Cysteine and methionine metabolism; Arginine and proline metabolism
Perillic acid	C_10_H_14_O_2_	166.099	up	1.445	0.017	5.516	Limonene and pinene degradation
Eicosapentaenoic acid	C_20_H_30_O_2_	308.235	up	1.576	0.014	6.040	Biosynthesis of unsaturated FAs
Docosahexaenoic acid	C_22_H_32_O_2_	328.236	up	1.624	0.020	4.988	Biosynthesis of unsaturated FAs
d-Erythrose 4-phosphate	C_4_H_9_O_7_P	200.008	down	1.703	0.004	0.379	Pentose phosphate pathway; Biosynthesis of amino acids; Vitamin B6 metabolism; Phenylalanine, tyrosine and tryptophan biosynthesis; Carbon metabolism; Microbial metabolism in diverse environments
Adenosine diphosphate ribose	C_15_H_23_N_5_O_14_P_2_	559.072	down	1.302	0.040	0.330	Purine metabolism
Cytidine	C_9_H_13_N_3_O_5_	243.086	down	1.467	0.030	0.555	Pyrimidine metabolism
Carnosine	C_9_H_14_N_4_O_3_	226.107	down	1.599	0.007	0.154	Histidine metabolism; beta-Alanine metabolism
l-Kynurenine	C_10_H_12_N_2_O_3_	208.085	down	1.269	0.047	0.573	Tryptophan metabolism
Syringic acid	C_9_H_10_O_5_	198.052	down	1.347	0.032	0.602	Aminobenzoate degradation; Microbial metabolism in diverse environments
Bisphenol A	C_15_H_16_O_2_	228.116	down	1.319	0.038	0.474	Microbial metabolism in diverse environments

Note: Metabolite identities are putative spectral matches from mzCloud and in-house libraries (mzVault/MassList) without authentic standard confirmation. Compounds, including bisphenol A, eicosapentaenoic acid and docosahexaenoic acid, are not *de novo* synthesized by *Enterobacterales* and may originate from culture media, laboratory contaminants or unresolved isomers.

**Fig. 2.**
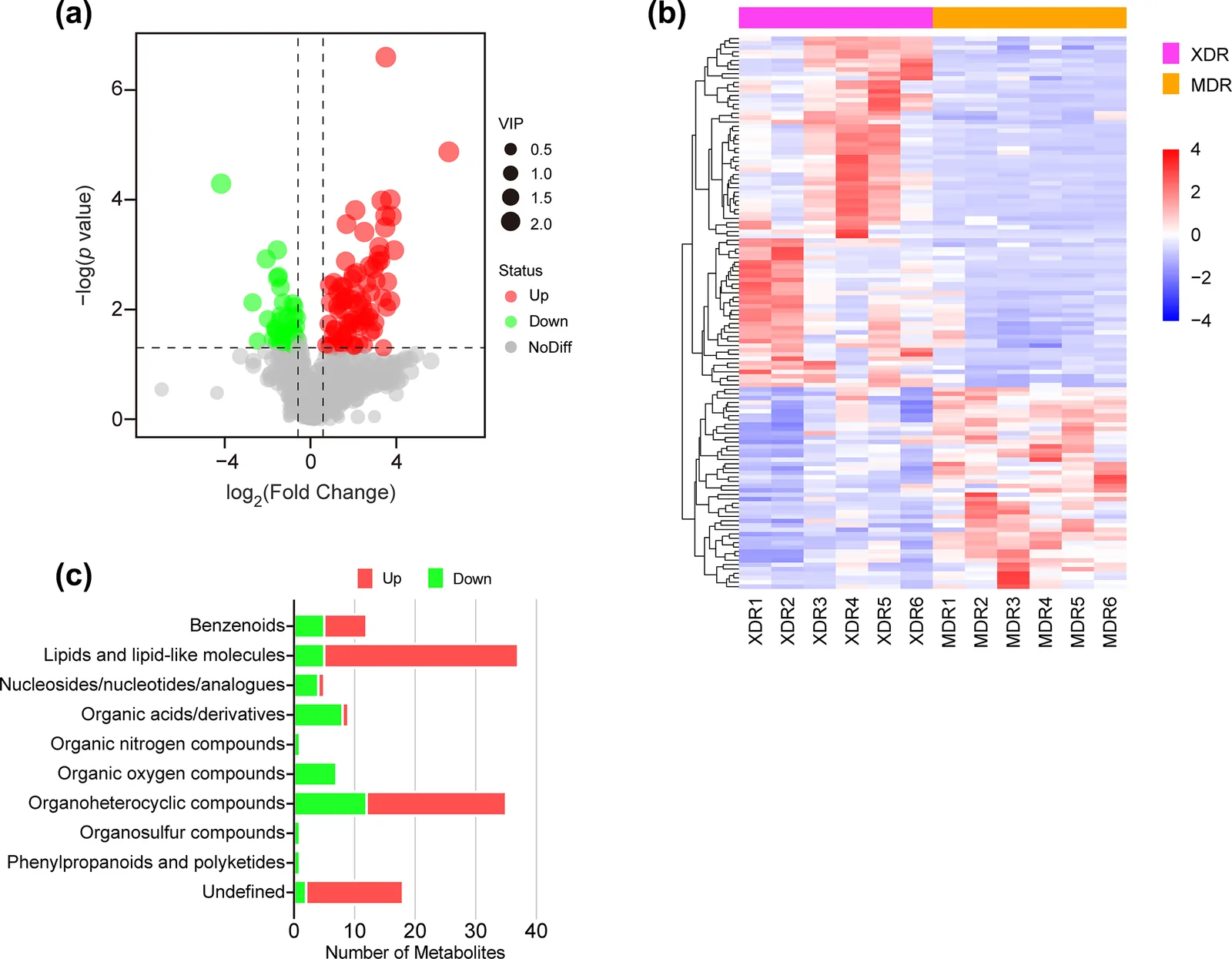
Analysis of DEMs between the XDR and MDR strains. (**a**) Volcano plot of the DEMs between colistin-resistant and -susceptible *K. pneumoniae*. The diameter of each dot was determined by its VIP value. (**b**) Heatmap profiles of the DEMs. (**c**) Classification information of the DEMs based on the ClassyFire database. Upregulated and downregulated metabolites were shown in red and green, respectively.

### Analysis of the lipid metabolism in the colistin-resistant *K. pneumoniae*

As illustrated in Fig. 2c, a number of lipids and lipid-like molecules were significantly disturbed in the colistin-resistant XDR strains. We further analysed the alterations of glycerophospholipids (GPLs), sphingolipid (SP) and fatty acids (FAs), which are important components of cell membrane and are crucial for bacterial survival [30]. As shown in Fig. 3, significantly altered GPLs, including phosphatidylethanolamine (PE), lysophosphatidylethanolamine (LPE) and lysophosphatidic acid (LPA), were up-regulated in the XDR strains. In addition, the levels of sphingosine (d18:1) and several FAs and conjugates were substantially elevated in the XDR strains. These lipid changes are consistent with possible membrane-associated adaptation in colistin-resistant isolates, although confirmation by gene-expression and functional analyses is required.

**Fig. 3.**
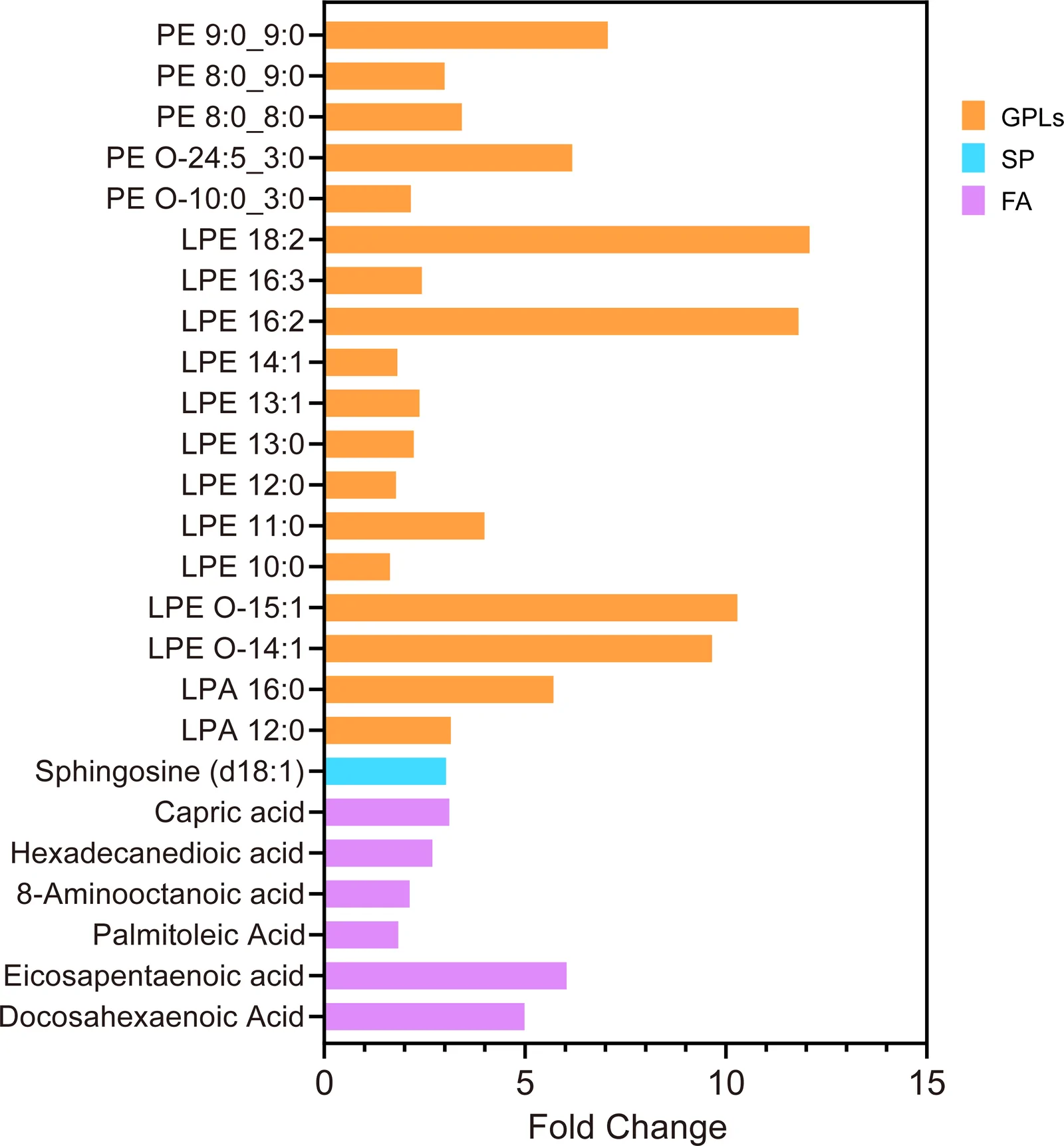
FCs of the significantly changed lipids in the colistin-resistant *K. pneumoniae*. PE, phosphatidylethanolamine; LPE, lysophosphatidylethanolamine; LPA, lysophosphatidic acid; GPLs, glycerophospholipids; SP, sphingolipid; FA, fatty acids and conjugates.

## Discussion

The global dissemination of CRKP poses a serious public health threat, which was markedly aggravated after the outbreak of COVID-19 [1, 2]. In this study, we integrated molecular profiling with untargeted metabolomics to investigate molecular and metabolic features associated with colistin resistance in clinical CRKP isolates. All examined strains belonged to the ST11 clone, a lineage frequently associated with hospital-acquired carbapenem resistance. Our findings demonstrated that colistin structure formed on biomaterial resistance in these isolates was chromosomally mediated, primarily through mutations in *pmrB* and *mgrB*, with no plasmid-encoded *mcr* genes detected. Every isolate carried multiple virulence-associated genes, with K2 being the dominant capsular serotype. All exhibited biofilm-forming capacity, with colistin-resistant strains showing stronger biofilm formation. Untargeted metabolomics identified 126 DEMs between colistin-resistant and colistin-susceptible isolates, with a significant enrichment in lipids and lipid-like molecules. These changes included upregulated FAs, GPLs and SPs, coupled with downregulated metabolites of central carbon and amino acid pathways, underscoring metabolic reprogramming correlated with the colistin-resistant phenotype. Together, these findings delineated a multilayered resistance mechanism in colistin-resistant CRKP that integrated genetic mutations and metabolic reprogramming, highlighting the need for combination therapies targeting these complementary pathways.

Colistin resistance primarily arises from modifications to its bacterial target, lipopolysaccharide (LPS), which reduce drug binding [6]. These modifications are mediated by chromosomal two-component systems, particularly PmrA/PmrB and PhoP/PhoQ, which regulate LPS modification enzymes [9, 10]. Specifically, the PmrB sensor kinase detects environmental signals and activates PmrA, which in turn upregulates genes responsible for adding cationic groups to lipid A [9]. MgrB, a negative regulator of PhoP/PhoQ, becomes inactivated, causing constitutive pathway activation and further LPS modification [10]. In the present study, these chromosomal regulatory elements were the principal drivers of colistin resistance in ST11 CRKP isolates. We identified a specific point mutation in *pmrB* (D150Y), resulting in constitutive activation of the PmrA/PmrB system. Furthermore, insertional inactivation of the *mgrB* gene by IS5-like elements or nonsense mutations within *mgrB* disrupted its inhibitory function on the PhoP/PhoQ system. These findings align with a prior study where *mgrB* alterations were the primary mechanism of colistin resistance, accounting for 75% of resistant *K. pneumoniae* isolates from Iran [31]. While *pmrB* mutations were also commonly detected, complementation experiments confirmed *mgrB* alterations directly restored resistance, whereas *pmrB* mutations alone did not [31]. The absence of plasmid-mediated *mcr* genes in these clinical isolates underscores the dominance of these chromosomally mediated pathways in the development of colistin resistance within our hospital setting, highlighting the critical role of ongoing genomic surveillance in tracking the evolution of local resistance mechanisms.

Virulence factors play a crucial role in colonization, invasion and pathogenicity of *K. pneumoniae* [32]. The capsular polysaccharide, a key virulence factor, confers resistance to host phagocytosis and facilitates infection dissemination [32, 33]. In the current study, half of the examined strains (6/12) were of the K2 serotype, a classic hypervirulent capsular type [33]. All isolates belonged to the ST11 high-risk clone and produced carbapenemases, demonstrating significant biofilm-forming capacity. Biofilms, bacterial community structures formed on biomaterial surfaces, confer elevated resistance, leading to *K. pneumoniae* exhibiting substantially higher resistance to colistin than planktonic cells [29, 34]. Consistent with these findings [29, 34], our colistin-resistant XDR strains displayed particularly robust biofilm formation. Remarkably, although all K2 strains carried multiple virulence genes, they exhibited a negative string test for hypervirulent mucoid phenotype. This suggests an incomplete concordance between virulence gene carriage and phenotypic expression. Our findings align with global research demonstrating that K2 strains can combine with various clones (e.g. ST65, ST43, ST147) and carry resistance genes, such as KPC, NDM and even *mcr-1*, highlighting their remarkable plasticity in transcending genetic backgrounds and coexisting with multiple drug resistance genes [19, 35–37]. Consequently, K2-capsule *K. pneumoniae* has evolved to possess both classic hypervirulence (including strong biofilm formation) and MDR. The combination of robust biofilm-forming capacity with the ST11 high-risk clone further enhances its persistence in healthcare environments, significantly increases treatment difficulty and poses serious challenges for clinical infection control.

Metabolomic analysis provided further insights into the adaptive mechanisms of colistin-resistant strains. Our metabolomic data revealed a significant upregulation of key GPLs, specifically PE, LPE and LPA, in the colistin-resistant XDR strains. PE is a major constituent of the bacterial outer membrane [30]. Several studies have reported upregulated PE in colistin-resistant Gram-negative bacteria [34, 38]. Its elevated level may contribute to hepta-acylation of lipid A, forming a protective outer membrane barrier that strengthens LPS and reduces host immune recognition [38]. LPE and LPA are key precursors and signalling molecules in phospholipid biosynthesis pathways [39, 40]. Their elevated levels suggest an accelerated turnover and reorganization of the membrane lipids. This remodelling likely aims to create a membrane architecture that is more rigid or less permeable, further hindering the penetration of colistin [41, 42]. In addition, the marked enrichment of SPs and FAs suggests an adaptation of the cell membrane that is essential for maintaining outer membrane integrity and bacterial survival [30]. Consistent with previous findings [34], our study also demonstrated significant upregulation of SPs and multiple FAs/conjugates in colistin-resistant XDR strains. SPs contribute to membrane stability and may function as signalling molecules that regulate bacterial stress responses [43], while increased levels of specific FAs, particularly cyclopropane FAs and trans-unsaturated FAs, can enhance membrane rigidity and reduce permeability to antimicrobial agents [44]. Although functional genetic validation (e.g. qPCR or gene knockout) was not performed in this study, our previous quantitative proteomic analysis of a parallel cohort of ST11 CRKP isolates collected within our hospital identified significant upregulation of the ArnBCADT operon enzymes and PmrD at the protein level in colistin-resistant strains [14]. These proteins are directly responsible for the addition of l-Ara4N to lipid A, which reduces colistin binding. The consistency between our previous proteomic findings (functional enzyme upregulation) and the current metabolomic data (accumulation of membrane lipid metabolites) provides independent, multilayered evidence supporting the metabolic reprogramming associated with colistin resistance. This coordinated upregulation of diverse lipid species suggests a comprehensive membrane remodelling strategy in colistin-resistant strains, whereby accumulated GPLs and SPs/FAs collectively contribute to a less permeable cell envelope that hinders colistin penetration.

Metabolomic profiling further revealed a significant suppression of metabolites linked to central carbon metabolism, including d-erythrose 4-phosphate (a critical intermediate in the pentose phosphate pathway) and ADP-ribose, which plays roles in energy metabolism and stress-related signalling. This metabolic downturn may indicate a systemic recalibration of cellular resources in response to colistin-induced stress [45]. By potentially dampening central metabolic fluxes, the cell can divert energy and carbon skeletons away from growth-oriented processes and towards the intensive synthesis of protective membrane components like PE, LPE and LPA. Such metabolic reprogramming, which sacrifices growth for defence, has been increasingly recognized as a complementary and potent mechanism facilitating bacterial adaptation and survival against antimicrobial agents [46]. This is consistent with findings that bacteria can undergo extensive lipidome adaptation, including the biosynthesis of novel lipids, under nutrient stress or other challenging conditions to ensure survival [47].

Despite these insights, our study has several limitations. First, the sample size was relatively small, and all isolates were from a single medical centre, which may limit the generalizability of the findings and increase the risk of false discovery in untargeted metabolomic analysis. Second, the six XDR isolates harboured distinct colistin resistance-driving mutations (*pmrB* D150Y, a nonsense *mgrB* mutation and IS5-like *mgrB* insertions), indicating they are not clonally identical. Nevertheless, because we did not perform whole-genome sequencing and subsequent core-genome MLST, we cannot completely rule out subtle genetic relatedness or a narrow local outbreak clone; consequently, the metabolomic differences we observed might partly reflect clonal background rather than a universal resistance signature. Whole-genome sequencing, coupled with Kleborate and Kaptive for accurate clonal and capsular typing, will be essential in the future to resolve this issue. Furthermore, the functional validation of the identified metabolic changes through genetic or enzymatic assays was not performed, and future studies should aim to confirm the causal relationship between these metabolic shifts and colistin resistance.

## Conclusion

This exploratory study identified genetic and metabolic features associated with colistin resistance among ST11 CRKP isolates from a single medical centre. Colistin-resistant isolates showed alterations in *pmrB* and *mgrB* and displayed distinct metabolomic profiles characterized mainly by increased membrane-associated lipid metabolites. These parallel alterations raise the hypothesis that cell envelope remodelling and resistance-associated mutations may be part of a coordinated adaptive response, though the present data do not establish a causal link. Because the study is limited by a single-centre design, a small sample size and the absence of functional validation, our conclusions remain hypothesis-generating and correlative rather than definitive mechanistic statements. We cannot exclude the possibility that the observed metabolic shifts partly reflect local clonal background rather than universal resistance signatures. Moreover, these findings must be confirmed in larger, independent collections of ST11 CRKP isolates from diverse geographic settings. Nonetheless, the co-carriage of resistance and virulence traits in these dominant clones underscores the clinical urgency for enhanced surveillance. Future studies incorporating whole-genome sequencing, transcriptional analysis and genetic complementation are required to establish a causal relationship and to determine whether these metabolic pathways represent viable therapeutic targets.

## Supplementary material

10.1099/jmm.0.002217 Supplementary Material 1.

10.1099/jmm.0.002217 Supplementary Material 2.

## Abbreviations

APB: 3-aminophenylboronic acid
CLSI: Clinical and Laboratory Standards Institute
COVID-19: coronavirus disease 2019
CRKP: carbapenem-resistant *Klebsiella pneumoniae*
DEM: differentially expressed metabolite
FA: fatty acid
FC: fold change
GPL: glycerophospholipid
KEGG: Kyoto Encyclopedia of Genes and Genomes
LPA: lysophosphatidic acid
LPE: lysophosphatidylethanolamine
MDR: multidrug resistance
MLST: Multilocus sequence typing
PE: phosphatidylethanolamine
QC: quality control
SP: sphingolipid
ST: sequence type
UHPLC: ultra-high performance liquid chromatography
VIP: variable importance in the projection
XDR: extensively drug-resistant
