# Supplementary material for: Molecular and metabolic features of colistin-resistant ST11 carbapenem-resistant Klebsiella pneumoniae
Source: J Med Microbiol. 2026 Nov 3;75(11):002217. doi: 10.1099/jmm.0.002217 (PMC13730195; doi:10.1099/jmm.0.002217)
Supplement: Supplementary file 1 [file jmm-75-02217-s001.pdf]

Supplementary Figures

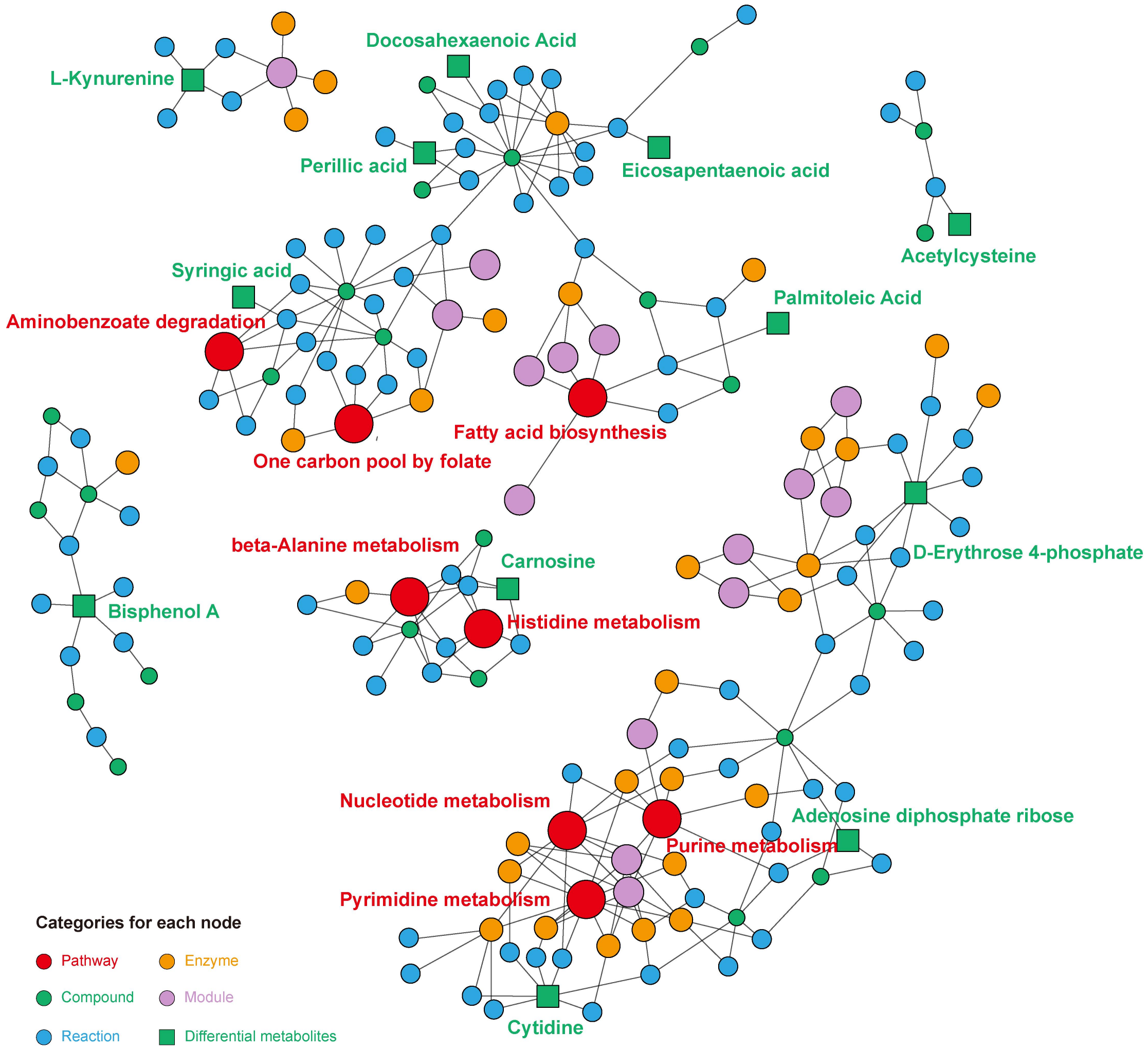

Figure S1. Metabolic regulation network analysis of DEMs between the XDR and MDR strains.
